# Different Approaches to Atopic Dermatitis by Allergists, Dermatologists, and Pediatricians

**DOI:** 10.1155/2021/6050091

**Published:** 2021-12-03

**Authors:** Suzieni Padoin Zuccolo de Bortoli, Herberto José Chong Neto, Nelson Augusto Rosário Filho

**Affiliations:** ^1^Federal University of Parana, Parana, Brazil; ^2^Pediatric Allergy and Immunology, Federal University of Parana, Parana, Brazil

## Abstract

**Objectives:**

Atopic dermatitis (AD) is the most prevalent chronic inflammatory skin disease, with a vast drug arsenal and guidelines available for its management and diagnosis and different medical specialties engaged in providing care. This study aimed to outline the therapeutic and diagnostic approaches to the AD of allergists, dermatologists, and pediatricians and verify whether they are compliant with the guidelines.

**Methods:**

A cross-sectional study using an electronic questionnaire administered through the SurveyMonkey® platform was disclosed by participating medical societies to their medical associates.

**Results:**

Of the 1,473 participating physicians, the use of moisturizers as part of AD treatment was observed among pediatricians (91.9%), dermatologists (97.5%), and allergists (100%; *p*=0.07). The preference for the use of new emollients was lower among pediatricians (57%) than dermatologists (75.9%) and allergists (71.4%; *p* < 0.001). The prevalence of wet-wrap therapy was lower among dermatologists (16.3%) than allergists (51%; *p* < 0.001). The recommendation of proactive treatment with topical corticosteroids was more frequently reported by allergists (65.3%) than pediatricians (43.3%) and dermatologists (40.8%; *p* < 0.001), and the same trend was observed in relation to proactive treatment using calcineurin inhibitors. The use of oral anti-histamines to control pruritus was mainly considered by pediatricians (69.2%) and dermatologists (59.2% *p* < 0.001). Clinical experience with systemic immunomodulating agents was greater among allergists (77.5%) and dermatologists (60.8%; *p* < 0.001), with cyclosporine being the most cited systemic immunomodulating agent. Environmental control of aeroallergens was recommended by pediatricians (89.8%), dermatologists (86.9%), and allergists (100%; *p*=0.01).

**Conclusion:**

There were differences in the therapeutic and diagnostic approaches to AD used by allergists, dermatologists, and pediatricians and those recommended by the guidelines, especially regarding the use of wet-wrap therapy, proactive treatment with topical corticosteroids or calcineurin inhibitors, prescription of anti-histamines, recommendation of phototherapy, and control of aeroallergens.

## 1. Introduction

Atopic dermatitis (AD) or atopic eczema is the most prevalent chronic inflammatory skin disease worldwide [1–3], and it is estimated that 20% to 30% of children and 7% to 10% of adults are affected [4]. Its heterogeneous and multifactorial etiology encompasses epithelial barrier dysfunction, immune dysregulation, changes in the skin microbiome, and genetic and environmental factors [5]. The increase in its occurrence observed in recent decades [6] represents a reason for concern in terms of global public health [7, 8]. The disease evolves in cycles of exacerbations, exhibits an allergic and hereditary nature, and frequently occurs in families with a history of other allergic diseases, such as asthma, allergic rhinitis, and allergic conjunctivitis [9].

Diagnosis is made using clinical criteria [10]. Pruritus, its most striking symptom in addition to skin xerosis, skin redness and inflammation [11], and consequent sleep disorders [12], has a strong negative impact on the quality of life of its bearers, affecting their physical appearance and causing psychological, psychosocial, and occupational disorders [10, 11, 13].

The main objective of therapeutic management is to control these signs and symptoms to provide relief and ensure a better quality of life [14–16]. For this, there is a wide range of therapies, multidisciplinary care [17], and guidelines published by Brazilian and international medical societies that are derived from expert consensus based on available scientific data [9, 15, 18–25]. Adherence to the guidelines does not ensure successful treatment in every situation [21], but it can help physicians make decisions grounded in evidence-based medicine in daily clinical practice.

Advances have been made in understanding the pathophysiology of AD and in the development of better-targeted therapies [26, 27]; however, little is known about the therapeutic decision-making of specialist physicians [28–30].

In this context, this study aimed to outline the therapeutic and diagnostic approaches to AD used by allergists, dermatologists, and pediatricians and verify whether they are compliant with the guidelines.

## 2. Methods

This was a cross-sectional study performed using the SurveyMonkey® electronic platform (EUA, 1999). The questionnaire was administered to Brazilian allergists, dermatologists, and pediatricians. The questionnaire was prepared by the authors and consisted of 34 questions divided into 2 sections: (1) sociodemographic data: gender, age, state of professional activity, academic background, specialist title or specialist registration with the Regional Medical Council, length of professional experience, and location of professional activity and (2) treatment of AD in adult and pediatric patients, with 30 questions addressing the use of moisturizers, emollients, wet-wrap therapy, corticosteroids, calcineurin inhibitors, anti-histamines, phototherapy, the role of superantigens, immunomodulatory agents, type of research performed, and dietary and environmental guidelines (Appendix 1).

The questionnaire was based on the updated guidelines of the Brazilian Association of Allergy and Immunology (BAAI; *Associação Brasileira de Alergia e Imunologia* – ASBAI); Brazilian Society of Pediatrics (BSP; *Sociedade Brasileira de Pediatria* – SBP); Brazilian Society of Dermatology (BSD; *Sociedade Brasileira de Dermatologia* – SBD); American Academy of Dermatology (AAD); European Academy of Dermatology and Venereology (EADV); Joint Task Force on Practice Parameters (JTF), representing the American Academy of Allergy, Asthma & Immunology (AAAAI); the American College of Allergy, Asthma, and Immunology (ACAAI); and the Joint Council of Allergy, Asthma, and Immunology. The questionnaire included aspects related to the following topics: (a) topical treatment of AD (hydration, corticosteroids, immunomodulators, and anti-histamines), (b) treatment with phototherapy and systemic agents of AD (phototherapy, systemic corticosteroids, oral anti-histamines, azathioprine, cyclosporine, interferon gamma, methotrexate, mycophenolate mofetil, dupilumab, and omalizumab), and (c) triggering factors (aeroallergens and food allergens).

The survey was distributed through participating medical societies, including the BAAI, BSD, and BSP, to their members by e-mail. The e-mail contained the link to access the questionnaire and was sent between April 2019 and February 2020.

Stratified random sampling was performed, and the proportions between the specialties, allergists, dermatologists, and pediatricians, were set considering the Brazilian medical demography reference population of 1,601 allergists, 8,137 dermatologists, and 39,234 pediatricians. The indicated sample was 3% of each tear with a 2.5% margin of error and a 95% trust level.

**Figure 1:**
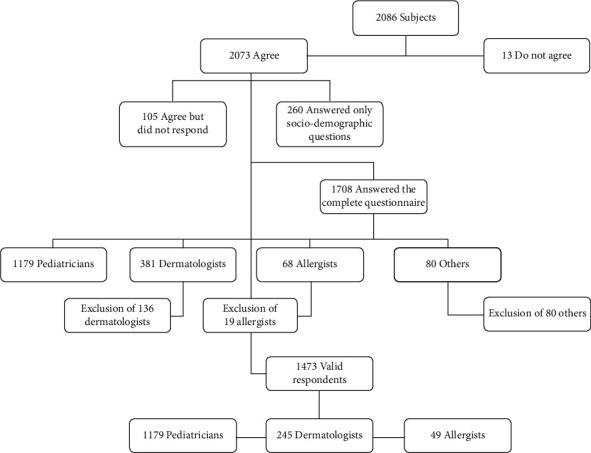
Flowchart of subjects.

The total number of questionnaires answered was 2,086, of which 1,708 (89.9%) questionnaires were completely answered. Of these, 80 were excluded for not having indicated a specialty, and 155 were excluded by random drawing to respect the proportionality of the population by specialty (3%). The final sample consisted of 1,473 questionnaires completed by 49 allergists, 1,179 pediatricians, and 245 dermatologists (Figure 1).

The difference between frequencies was studied through contingency tables using Pearson's chi-squared test, considering a significance level of 5% (Statistica® 7.0 Statsoft).

## 3. Results

Among the 1,473 eligible questionnaires, 1,179 were answered by pediatricians (80%), 245 by dermatologists (16.7%), and 49 (3.3%) by allergists. The questionnaires were predominantly completed by female professionals aged between 30 and 60 years who were active, especially in southeastern and southern Brazil, mainly in private practice offices (Table 1).

Regarding the use of moisturizers, 1,167 of the pediatricians (99%), 244 of the dermatologists (99.6%), and 49 of the allergists (100%) indicated that the use of moisturizers can reduce the severity of AD (*p*=0.96), and 1,083 (91.9%), 239 (97.5%), and 49 (100%), respectively, reported prescribing moisturizers as an integral part of AD treatment (*p*=0.07). The preference for the use of new emollients, which influence the skin microbiome, was lowest among pediatricians (672 (57%) vs. 186 dermatologists (75.9%) and 35 allergists (71.4%); *p* < 0.001). Regarding the prescription of wet-wrap therapy, namely, moist compresses with and without topical corticosteroids for moderate or severe AD, a lower frequency was also observed among pediatricians (319 (27%)) and dermatologists (40 (16.3%)), but 25 allergists reported prescribing this treatment (51%; *p* < 0.001). A total of 511 pediatricians (43.3%), 100 dermatologists (40.8%), and 32 allergists (65.3%; *p* < 0.001) responded positively to the recommendation of topical corticosteroids for the prevention of recurrence (proactive treatment) in patients with recurrent crises in the same body locations. In addition, 268 pediatricians (22.7%), 128 dermatologists (52.2%), and 29 allergists (59.2%) responded positively to proactive treatment with calcineurin inhibitors, and the highest rates were noted for allergists (*p* < 0.001). As a second-line treatment, calcineurin inhibitors, especially for sensitive areas, were also more frequently considered by allergists (47 (95.9% vs. 848 pediatricians (71.9%) and 209 dermatologists (85.3%; *p* < 0.001; Table 2).

Of the participants, 268 pediatricians (22.7%), 19 dermatologists (7.8%), and 6 allergists (12.2%) reported preferring topical corticosteroids instead of calcineurin inhibitors for the treatment of recurrent inflammatory lesion (*p* < 0.001), and 165 pediatricians (14%), 33 dermatologists (3.5%), and 6 allergists (12.2%) indicated that they usually prescribe topical anti-histamines. Four hundred and twenty-seven pediatricians (36.2%), 92 dermatologists (37.5%), and 20 allergists (40.8%) reported the use of oral corticosteroids as the first line of systemic therapy when topical use was not effective. In addition, 1,121 pediatricians (95.1%), 240 dermatologists (98%), and all allergists (100%) agreed that systemic corticosteroids were not recommended for children with AD as a long-term therapy, whereas only 67 pediatricians (5.7%), 10 dermatologists (4.1%), and 3 allergists (6.1%) answered that they maintained long-term treatments with systemic corticosteroids (*p*=0.95) in the absence or unavailability of other therapies (Table 2).

The use of phototherapy as the next treatment option after basic topical treatment failed for moderate or severe AD was higher among dermatologists (131 (53,5%)) compared with pediatricians (463 (39.3%)) and allergists (11 (22.4%);*p* < 0.001). The use of oral anti-histamines to control pruritus was considered mainly by pediatricians (816 (69.2%)) and dermatologists (145 (59.2%)) and was less frequently prescribed by allergists (17 (34.7%); *p* < 0.001; Table 2).

The role of superantigens in AD was recognized by allergists (46 (93.9%)) and dermatologists (185 (75.5%)) but to a lesser extent among pediatricians (313 (26.5%); *p* < 0.001). Allergists also most frequently reported the use of therapeutic measures for its control (41 (83.7% vs. 111 dermatologists (45.3%) and 208 pediatricians (17.6%); *p* < 0.001; Table 2).

There was no significant difference in the frequency of prescription of oral anti-histamines (653 pediatricians (55.4%), 144 dermatologists (58.8%), and 31 allergists (63.3%); *p*=0.32)). Clinical experience with systemic immunomodulatory agents was more frequent among allergists (38 (77.5%)) and dermatologists (149 (60.8%)) compared with pediatricians (197 (16.7%); *p* < 0.001). Cyclosporine was the most cited systemic immunomodulatory agent (318 pediatricians (27%), 114 dermatologists (46.5%), and 32 allergists (65.3%)) with the highest frequency of prescription noted in the last two categories of professionals (*p* < 0.001; Table 2).

Dupilumab was considered a therapeutic option due to its availability in the Brazilian pharmaceutical market, especially by allergists (35 (71.4% vs. 240 (20.4%) pediatricians and 101 (41.2%) dermatologists; *p* < 0.001). Investigations of the relationship between AD and food allergies were considered by 683 pediatricians (57.9%), 57 dermatologists (23.3%), and 27 allergists (55.1%; *p* < 0.001), and lgE research was performed by 859 pediatricians (72.8%), 117 dermatologists (47.7%), and all allergists (100%; *p* < 0.001; Table 2).

Dietary restrictions in AD patients were reported by 560 pediatricians (47.5%), 84 dermatologists (34.3%), and 19 allergists (38.8%; *p* < 0.001). Moreover, the implementation of dietary restrictions based on positive lgE-specific allergy/research skin tests, which is consistent with a clinical history of cause and effect, was considered by 939 pediatricians (79.6%), 148 dermatologists (60.4%), and 44 allergists (89.8%; *p* < 0.001; Table 2).

Environmental control of aeroallergens was observed among 1,059 pediatricians (89.8%), 213 dermatologists (86.9%), and all allergists (100%; *p* < 0.01).

All professional categories reported investigating immunodeficiencies in patients with moderate/severe AD with the highest rates noted for allergists (31 (63.3%) vs. 593 pediatricians (50.3%) and 75 dermatologists (30.6%); *p* < 0.001; Table 2).

At least one complimentary test to investigate immunodeficiencies in patients with moderate/severe AD was indicated by 1,092 pediatricians (92.6%), 192 dermatologists (78.4%), and all allergists (100%; *p* < 0.01; Table 2).

## 4. Discussion

In recent years, considerable progress has been made in understanding the etiopathogenesis of atopic dermatitis. The main triggering and/or aggravating agents have been identified, and new perspectives with the application of precision medicine and emerging therapeutics have brought encouraging results [31]. Recent studies indicate a substantial increase in the prevalence of AD [32] and point to its complexity related to genetic predisposition, phenotypic and molecular variations, and immune status. Early diagnosis and treatment are essential, and the choice of therapeutic strategies must consider the clinical and individual variability of the disease [33]. Among the therapeutic measures evaluated, the use of moisturizers was a consensus among experts with the aim of reducing the severity of AD and the use of medication. The AD guidelines are equally unanimous in recommending the use of moisturizers to promote the restoration of the skin barrier, reduce transepidermal water loss, improve xerosis, and decrease the signs and symptoms of AD, including pruritus, erythema, fissure, and lichenification. Furthermore, the consistent use of moisturizers decreases the amount of topical anti-inflammatory agents needed to control the disease, especially those moisturizers referred to as advantageous due to the sparing effect of topical corticosteroids. To ensure adequate hydration, generous and frequent use of moisturizers is suggested [9, 18, 20, 21, 25].

Regarding new emollients, differences are noted among the guidelines. EADV guidelines refer to new emollients as “emollients plus,” which are topical formulations composed of a moisturizing vehicle plus nondrug active ingredients. Such active ingredients accelerate the recovery of the skin barrier and the growth of commensal bacteria to recover the diversity of the skin microbiome, supporting the beneficial effects and protective role of the skin microbiota in skin defenses [9]. The BAAI/BSP, EADV, and BSD guidelines discuss the benefit of these “plus” emollients with active ingredients, such as saponins, flavonoids, riboflavin, and bacterial lysates from *Aquaphilus dolomiae* or *Vitreoscilla filiformis*. Although recognized, there is no official recommendation for the adoption of these emollients to the detriment of standard moisturizers [9, 18, 20].

Greater than 60% of the responding professionals reported adhering to this therapeutic measure, indicating that these emollients seem promising in clinical practice.

Regarding wet-wrap therapy, although unanimously recommended to rapidly reduce the severity of the disease in situations of significant crises or even in cases of recalcitrant disease [9, 18, 20, 21, 25], approximately 50% of the participants indicated never or almost never prescribing this therapy.

The use of topical corticosteroids as a first-line anti-inflammatory treatment in adults and children with AD to rescue the patient from inflammatory crises when the moisturizer alone is unable to control the disease [9, 18, 20, 21, 25] is consistently suggested by all guidelines and was reported by most of the participants instead of calcineurin inhibitors. Maintenance therapy or proactive therapy with the intermittent application once or twice a week in specific locations is recommended by all analyzed guidelines and is more effective than the use of emollients alone to reduce relapses and the severity of AD. In total, 44% of the participants reported adhering to this practice.

Topical calcineurin inhibitors, which are also called topical immunomodulators and include pimecrolimus and tacrolimus, are the second-line anti-inflammatory therapy unanimously recommended by the guidelines for adults and children with AD. Topical calcineurin inhibitors are especially valuable in sensitive areas (face, intertriginous places, and anogenital area) because they do not induce skin atrophy. Proactive therapy, which consists of twice a week application of tacrolimus ointment combined with moisturizers to previously affected areas to help prevent relapses, was proposed in AAD, EADV, JTF, and BSD guidelines [9, 18, 21, 22, 25]. The BAAI/BSP does not specifically address the issue, whereas the AAD recommends that these agents can be proactively used two to three times a week and that the practice can reduce the need for topical corticosteroids. In addition, this combination is more effective than the use of emollients alone [21, 22]. Unlike other guidelines, AAD guidelines suggest concomitant therapy with topical calcineurin inhibitors and topical corticosteroids [21, 22]. This was a point of divergence as greater than 42% of the participants never or almost never recommended the proactive use of calcineurin inhibitors.

The use of topical anti-histamines in the treatment of pruritus has minimal utility with no significant reduction in the severity or disease control or in the potential for skin sensitization. The use of topical anti-histamines is not recommended in the AAD, EADV, and JTF guidelines [9, 21, 25] or even discussed in the BAAI/BSP and the BSD guidelines. However, approximately 40% of the participants still reported prescribing them.

Phototherapy is recommended as an adjuvant treatment for AD refractory to first-line treatment (emollients, topical steroids, and topical calcineurin inhibitors) [9, 18, 20, 24, 25]; however, almost 60% of respondents did not consider the use of phototherapy in this situation.

The consulted guidelines agree that systemic corticosteroids are not recommended for long-term use due to the unfavorable benefit-risk ratio associated with side effects and rebound, and this was notion was supported by greater than 95% of the participants. These drugs are useful only as quick courses in severe exacerbations in exceptional cases [15, 18, 20, 24, 25].

Satisfactory evidence to support the use of oral anti-histamines as an integral part of AD treatment for the relief of pruritus is lacking; however, this practice was mentioned by approximately 67% of the participants. Sporadic and short-term use of first-generation anti-histamines may be favorable in the setting of sleep loss secondary to pruritus but should not replace standard AD treatment with topical therapies. In addition, the sleep quality induced by such drugs is not ideal [9, 18, 20, 24, 25].

There is consensus among the considered guidelines when recommending cyclosporine for forms of AD that are severe and refractory to classical treatments [15, 18, 20, 24, 25]. The BAAI/BSP and EADV considered cyclosporine as a first-line treatment among the different options of systemic immunosuppressive agents used to treat AD [15, 20]. Notably, the BAAI/BSP and EADV suggest azathioprine as a second-line therapy when cyclosporine is not effective or contraindicated [15, 20], and azathioprine is indicated for moderate or severe disease [15, 18, 20, 24, 25]. Interferon gamma is recommended only by the AAD guidelines, which specifies its utility as an alternative therapy for refractory AD in adults and children who have not responded or have contraindications to the use of other systemic therapies or phototherapy [24] and JTF [24, 25]. The BAAI/BSP guidelines do not include this recommendation due to the availability of medications with a better cost per response ratio in addition to a more favorable safety profile [20]. Methotrexate is an AD treatment; however, specific safety recommendations must be observed [15, 18, 20, 24, 25]. For example, BSD indicates its use as an initial treatment in moderate to severe AD recalcitrant to treatment with topical corticosteroids in addition to highlighting its usefulness in long-term management [18]. The AAD recommends mycophenolate mofetil as an alternative systemic therapy for refractory AD [24]. On the other hand, the EADV specifies that its use should be restricted to adult patients if cyclosporine is not effective or not indicated [15], whereas the BAAI/BSP recommends it as a third-line therapeutic option [20]. Among the participants, most of the experts (74%) reported having no clinical experience with these systemic immunomodulating agents. Few reported having experience with the use of cyclosporine (19%) and slightly less than 17% note experience with methotrexate. However, these respondents considered cyclosporine as the first-line systemic treatment in the management of moderate to severe AD.

Dupilumab is the first immunobiologically approved treatment for AD. It is a fully human recombinant lgG4 monoclonal antibody that inhibits IL-4 and IL-13 signaling and reduces disease symptoms by inhibiting the inflammation process [34]. Dupilumab is approved to treat AD effectively and safely in patients aged 6 years and older [35]. Among the guidelines, the most recent guidelines from the BAAI/BSP, EADV, and the BSD do not yet recommend dupilumab for children but do highlight its effectiveness in the remission of signs and symptoms, including pruritus [15, 18, 20]. Greater than 25% of the experts revealed having a patient who is a candidate for this therapy.

The guidelines do not recommend omalizumab for the treatment of AD [15, 20, 24, 25], and the BSD guidelines do not specifically debate the subject.

The relation between AD and food allergies is a matter of great discussion in the literature [19, 36]. The guidelines recommend dietary restrictions based on allergic skin testing and specific IgE research consistent with a clinical history of cause and effect [9, 18, 19, 22, 25], and approximately 77% of experts agree with this recommendation.

The guidelines differ regarding the environmental control of aeroallergens for patients with AD. The BAAI/BPS and JTF [18, 31] guidelines note that approaches to reduce contact with aeroallergens should only be adopted in highly sensitive patients with moderate to severe chronic symptoms [19, 25], and most of the participants indicated this recommendation.

This research has limitations that primarily involve the type of data collected. Specifically, the data are self-reported and obtained from objective answers. Thus, these data cannot be verified independently. Other limitations include potential nonresponder bias, confirmation, anchoring, perception, and the halo effect, which occurs when a respondent has a positive view of something even before experiencing it; and the framing effect, which occurs when the selected response is affected by the way the question, is asked. These limitations are inherent to this type of inquiry.

AD is a debilitating, complex, and multifactorial systemic disease and one of the most prevalent chronic inflammatory skin conditions affecting pediatric and adult populations. Its study and comprehension are inspired by different perspectives, such as its epidemiology, etiopathogenesis, and therapeutic approach [27, 37].

There is research potential in the investigation of therapeutic approaches to AD due to ongoing advances in science and available therapy. Concerning treatment consensuses, adherence to such guidelines will not ensure the success of clinical treatment under any circumstances. These guidelines merely assist physicians by categorizing the available therapeutic options and providing critical details to better treat each patient. However, systematic analyses of such treatment consensuses can provide relevant findings.

In sum, the opinions converged on the use of moisturizers, prescription of topical and oral anti-histamines, and restricted use of systemic corticosteroids.

Dermatologists and allergists were more often in agreement regarding the use of new emollients and calcineurin inhibitors, recognition of the role of superantigens, and clinical experience with systemic immunomodulators.

Pediatricians and dermatologists were more often in agreement regarding the use of topical corticosteroids in proactive treatment, phototherapy, environmental restriction measures, and the effectiveness of oral anti-histamines in pruritus.

Pediatricians and allergists were more often in agreement regarding investigations of the relation between atopic dermatitis and food allergy, lgE and dietary restriction based on specific positive tests, and immunodeficiency.

There was disagreement among professionals regarding the use of wet-wrap therapeutic measures to control superantigens, which was more frequently used by allergists, and the preference for calcineurin over corticosteroids in inflammatory crises, which was more frequently noted among pediatricians.

## 5. Conclusion

Differences were noted between the therapeutic and diagnostic approaches of allergists, dermatologists, and pediatricians in AD in relation to those recommended by the guidelines, especially regarding adherence to wet-wrap therapy, proactive treatment with topical corticosteroids or calcineurin inhibitors, prescription of anti-histamines, recommendation of phototherapy, and control of aeroallergens. Such differences may be related to the fact that AD management must be individualized, adapted based on its clinical variability, and delivered with the main purpose of providing patients with adequate disease control.

## Figures and Tables

**Table 1:** Sociodemographic data according to the different specialties.

	Pediatricians (*n* = 1.179)	Dermatologists (*n* = 245)	Allergists (*n* = 49)	Total (*n* = 1.473)	*p*
Female gender	933 (79.1%)	213 (86.9%)	32 (65.3%)	1178 (80.0%)	0.03
Age (years)					
21 to 29	71 (6.0%)	25 (10.2%)	2 (4.1%)	98 (6.6%)	0.06^*∗*^
30 to 39	433 (36.7%)	106 (43.3%)	14 (28.6%)	553 (37.5%)	
40 to 49	254 (21.5%)	55 (22.4%)	11 (22.4%)	320 (21.7%)	
50 to 59	252 (21.4%)	32 (13.1%)	16 (32.6%)	300 (20.4%)	
>60	169 (14.3%)	27 (11.0%)	6 (12.2%)	202 (13.7%)	
Brazilian states					
North	61 (51.2%)	6 (2.4%)	0 (0.0%)	67 (4.5%)	0.17
Northeast	184 (15.6%)	33 (13.5%)	9 (18.4%)	226 (15.3%)	
Midwest	90 (7.6%)	16 (6.5%)	0 (0.0%)	106 (7.2%)	
Southeast	608 (51.6%)	147 (60.0%)	30 (61.2%)	785 (53.3%)	
South	236 (20.0%)	43 (17.5%)	10 (20.4%)	289 (19.6%)	
Academic training					
Medical residency	882 (74.8%)	138 (56.3%)	25 (51.0%)	1.045 (70.9%)	<0.01
Specialization	282 (23.9%)	86 (35.1%)	22 (44.9%)	390 (26.5%)	
Master's degree	170 (14.4%)	48 (19.6%)	11 (22.4%)	229 (15.5%)	
Doctorate	69 (5.8%)	15 (6.1%)	12 (24.5%)	96 (6.5%)	
Postdoctorate	7 (0.6%)	1 (0.4%)	4 (8.2%)	12 (0.8%)	
Practice time (years)					
<5	106 (9.0%)	24 (9.8%)	2 (4.1%)	132 (9.0%)	0.07^#^
5 to 9	236 (20.0%)	47 (19.2%)	11 (22.4%)	294 (20.0%)	
10 to 19	296 (25.1%)	86 (35.1%)	7 (14.3%)	389 (26.4%)	
20 to 29	257 (21.8%)	45 (18.4%)	11 (22.4%)	313 (21.2%)	
≥30	284 (24.1%)	43 (17.5%)	18 (36.7%)	345 (23.4%)	
Kind of work					
Private	820 (69.5%)	221 (90.2%)	45 (91.8%)	1.086 (73.7%)	<0.001
Polyclinic	99 (8.4%)	40 (16.3%)	3 (6.1%)	142 (9.6%)	
Public hospital	598 (50.7%)	63 (25.7%)	13 (26.5%)	674 (45.8%)	
Private hospital	472 (40.0%)	38 (15.5%)	10 (20.4%)	520 (35.3%)	
Primary health care unit	273 (23.2%)	22 (9.0%)	4 (8.2%)	299 (20.3%)	
University	226 (19.2%)	47 (19.2%)	20 (40.8%)	293 (19.9%)	
Others	82 (7.0%)	14 (5.7%)	2 (4.1%)	98 (6.6%)	

Pearson's cui-square test. ^*∗*^Comparison gathering 30 to 59 years old. ^#^Comparison gathering 5 to 29 years old.

**Table 2:** Questionnaire answers (always and almost always).

Questions	Pediatricians (*n* = 1.179)	Dermatologists (*n* = 245)	Allergists (*n* = 49)	Total (*n* = 1.473)	*p*
1^1^	1167 (99.0%)	244 (99.6%)	49 (100.0%)	1460 (99.1%)	0.96
2^2^	1083 (91.9%)	239 (97.5%)	49 (100.0%)	1371 (93.1%)	0.07
3^3^	672 (57.0%)	186 (75.9%)	35 (71.4%)	893 (60.6%)	<0.001
4^4^	319 (27.0%)	40 (16.3%)	25 (51.0%)	384 (26.0%)	<0.001
5^5^	511 (43.3%)	100 (40.8%)	32 (65.3%)	643 (43.6%)	<0.001
6^6^	268 (22.7%)	128 (52.2%)	29 (59.2%)	425 (28.8%)	<0.001
7^7^	848 (71.9%)	209 (85.3%)	47 (95.9%)	1104 (74.9%)	<0.001
8^8^	268 (22.7%)	19 (7.8%)	6 (12.2%)	293 (19.9%)	<0.001
9^9^	165 (14.0%)	33 (13.5%)	6 (12.2%)	204 (13.8%)	0.84
10^10^	427 (36.2%)	92 (37.5%)	20 (40.8%)	539 (36.6%)	0.70
11^11^	1121 (95.1%)	240 (98.0%)	49 (100.0%)	1410 (95.7%)	0.19
12^12^	67 (5.7%)	10 (4.1%)	3 (6.1%)	80 (5.4%)	0.95
13^13^	463 (39.3%)	131 (53.5%)	11 (22.4%)	605 (41.1%)	<0.001
14^14^	816 (69.2%)	145 (59.2%)	17 (34.7%)	978 (66.4%)	<0.001
15^15^	313 (26.5%)	185 (75.5%)	46 (93.9%)	544 (36.9%)	<0.001
16^16^	208 (17.6%)	111 (45.3%)	41 (83.7%)	360 (24.4%)	<0.001
17^17^	653 (55.4%)	144 (58.8%)	31 (63.3%)	828 (56.2%)	0.32
18^18^	197 (16.7%)	149 (60.8%)	38 (77.5%)	384 (26.1%)	<0.001^a^
19^19^	318 (27.0%)	114 (46.5%)	32 (65.3%)	464 (31.5%)	<0.001^b^
20^20^	240 (20.4%)	101 (41.2%)	35 (71.4%)	376 (25.5%)	<0.001
21^21^	683 (57.9%)	57 (23.3%)	27 (55.1%)	767 (52.1%)	<0.001
22^22^	859 (72.8%)	117 (47.7%)	49 (100.0%)	1025 (69.6%)	<0.001
23^23^	560 (47.5%)	84 (34.3%)	19 (38.8%)	663 (45.0%)	<0.001
24^24^	939 (79.6%)	148 (60.4%)	44 (89.8%)	1131 (76.8%)	<0.001
25^25^	1059 (89.8%)	213 (86.9%)	49 (100.0%)	1321 (89.7%)	0.01
26^26^	593 (50.3%)	75 (30.6%)	31 (63.3%)	400 (27.1%)	<0.001
27^27^	1092 (92.6%)	192 (78.4%)	49 (100.0%)	1333 (90.5%)	<0.01^c^

Pearson's cui-square test. ^a^Yes to some immunomodulator, ^b^most frequent answer: cyclosporine, and ^c^ask for at least one laboratory test. ^1^Do you believe that the use of moisturizers can reduce the severity of AD? ^2^Do you prescribe moisturizer as an integral part of the treatment of atopic dermatitis? ^3^ Do you prefer the new emollients or “plus” emollients, which influence the cutaneous microbiome? ^4^Do you prescribe the use of “wet-wrap” therapy, wet compresses, with or without a topical corticosteroid, for patients with moderate or severe AD? ^5^For patients with recurrent crises in the same body sites, do you recommend topical corticosteroids for relapse prevention (proactive treatment)? ^6^Do you recommend the proactive use of calcineurin inhibitors as maintenance treatment (2 to 3 times per week)? ^7^Do you believe that calcineurin inhibitors are the second-line treatment, especially indicated for sensitive areas? ^8^Do you prefer calcineurin inhibitors over topical corticosteroids in crisis in inflammatory lesions? ^9^Do you prescribe topical anti-histamines for AD patients? ^10^When topical treatment is not sufficient, are oral corticosteroids your first choice for systemic treatment*?*^11^Do you agree that systemic corticosteroids are not recommended for children with atopic dermatitis, but only as a short-term transition to other therapies? ^12^Do you maintain long-term treatment with systemic corticosteroids in the absence of phototherapy or other unavailability therapies? ^13^In patients with moderate/severe and/or refractory AD, is phototherapy your next treatment option to basic topical treatment (moisturizers, topical corticosteroids, and/or calcineurin inhibitors)? ^14^Do you consider oral anti-histamines effective in controlling pruritus? ^15^Do you know the role of superantigens in AD? ^16^Do you use therapeutic measures for the control of superantigens? ^17^Do you prescribe oral anti-histamines for AD patients? ^18^Do you have clinical experience with any of the systemic immunomodulatory agents in atopic dermatitis? ^19^Which of these do you consider to be first-line treatment? ^20^With Dupilumab available in the Brazilian pharmaceutical market, do you have any patient to start this therapy? ^21^Do you usually investigate the association between atopic dermatitis and food allergy? ^22^Do you request specific IgE research for the suspected allergen by which method? ^23^Do you place dietary restrictions on AD patients? ^24^Are dietary restrictions based on positive allergy skin tests/specific IgE test and consistent with a clinical history of cause-and-effect relationship? ^25^Do you recommend environmental control of aeroallergens to AD patients? ^26^Do you investigate immunodeficiencies in patients with moderate/severe AD? ^27^What tests would you order to investigate immunodeficiencies in patients with moderate/severe AD?

## Data Availability

The data used to support the findings of this study are included within the article.

## Supplementary Materials

Supplementary Materials Appendix 1: atopic dermatitis management questionnaire, Treatment of Atopic Dermatitis (AD).
