# Supplementary material for: Different Approaches to Atopic Dermatitis by Allergists, Dermatologists, and Pediatricians
Source: Dermatol Res Pract. 2021 Dec 3;2021:6050091. doi: 10.1155/2021/6050091 (PMC8664543; doi:10.1155/2021/6050091)
Supplement: Supplementary Materials — Appendix 1: atopic dermatitis management questionnaire, Treatment of Atopic Dermatitis (AD). [file 6050091.f1.docx]

Appendix 1 - Atopic dermatitis management questionnaire

1. What is your gender?

( ) Female ( ) Male

2. What is your age?

( ) 21 a 29 years ( ) 30 a 39 years ( ) 40 a 49 years ( ) 50 a 59 years ( ) > 60 years

3. In which Brazilian state do you currently work?

( ) Acre ( ) Alagoas ( ) Amapá ( ) Amazonas ( ) Bahia ( ) Ceará ( ) Distrito Federal ( ) Espirito Santo ( ) Goiás ( ) Maranhão ( ) Mato Grosso ( ) Mato Grosso do Sul ( ) Minas Gerais ( ) Pará ( ) Paraíba ( ) Paraná ( ) Pernambuco ( ) Piauí ( ) Rio de Janeiro ( ) Rio Grande do Norte ( ) Rio Grande do Sul ( ) Rondônia ( ) Roraima ( ) Santa Catarina ( ) São Paulo ( ) Sergipe ( ) Tocantins

4. Select the alternative(s) that correspond to your academic training?

( ) Medical residency program ( ) Improvement or Specialization with workload equivalent to medical residency ( ) Master’s degree ( ) Doctoral degree ( ) Post-Doctoral ( ) Other

Which one?_____________________

5. Do you have a specialist title or registration with the CRM in any of these specialties? (Select one or more alternatives)

( ) Allergy and Immunology ( ) Dermatology ( ) Pediatrics

6. How long have you been working as a doctor?

( ) < 5 years ( ) 5-9 years ( ) 10-19 years ( ) 20-29 years ( ) > 30 years

7. Where do you work? (Select one or more alternatives)

( ) Private Practice ( ) Polyclinic ( ) Public Hospital ( ) Private Hospital ( ) Primary Health Care Unit ( ) University

( ) Other Which one?___________________

**Treatment of Atopic Dermatitis (AD)**

1. Do you believe that applying moisturizers can reduce the severity of AD?

( ) Yes ( ) No

2. Do you prescribe moisturizer as an integral part of the treatment of AD?

( ) Always ( ) Almost always ( ) Sometimes ( ) Almost never ( ) Never

Do you prefer the new emollients or "plus" emollients, which influence the skin microbiome?

( ) Yes ( )

3. Do you prescribe the use of "wet-wrap" therapy, wet compresses, with or without a topical corticosteroid, for patients with moderate or severe AD ?

( ) Always ( ) Almost always ( ) Sometimes ( ) Almost never ( ) Never

4. For patients with recurrent crises in the same body sites, do you recommend topical corticosteroids for relapse prevention (proactive treatment)?

( ) Always ( ) Almost always ( ) Sometimes ( ) Almost never ( ) Never

5. Do you recommend the proactive use of calcineurin inhibitors as maintenance treatment (2-3 times per week)?

( ) Always ( ) Almost always ( ) Sometimes ( ) Almost never ( ) Never

6. Do you believe that calcineurin inhibitors are the second-line treatment, especially indicated for sensitive areas?

( ) Yes ( ) No

7. Do you prefer calcineurin inhibitors over topical corticosteroids in crises in inflammatory lesions?

( ) Yes ( ) No

8. Do you prescribe topical antihistamines for AD patients?

( ) Always ( ) Almost always ( ) Sometimes ( ) Almost never ( ) Never

9. When topical treatment is not sufficient, are oral corticosteroids your first choice for systemic treatment?

( ) Yes ( ) No

10. Do you agree that systemic corticosteroids are not recommended for children with atopic dermatitis, but only as a short-term transition to other therapies?

( ) Yes ( ) No

11. Do you maintain long-term treatment with systemic corticosteroids in the absence of phototherapy or unavailability of other therapies?

( ) Yes ( ) No

12. In patients with moderate/severe and/or refractory AD, is phototherapy your next treatment option to basic topical treatment (moisturizers, topical corticosteroids and/or calcineurin inhibitors)?

( ) Yes ( ) No

13. Do you consider oral antihistamines effective in controlling pruritus?

( ) Yes ( ) No

14. Do you know the role of superantigens in AD?

( ) Yes ( ) No

15. Do you use therapeutic measures for the control of superantigens?

( ) Yes. Which one?____________________________________________ ( ) No

16. Do you prescribe oral antihistamines for AD patients?

( ) Always ( ) Almost always ( ) Sometimes ( ) Almost never ( ) Never

17. Do you have clinical experience with any of the systemic immunomodulatory agents in Atopic Dermatitis? (Select one or more alternatives)

( ) I don’t have clinical experience with these treatments ( )Azathioprine ( )Cyclosporin

( ) Immunoglobulin ( ) Gamma Interferon ( ) Methotrexate ( ) Mofetil mycophenolate

( ) Omalizumab ( ) Others

18. Which of these, do you consider to be first-line treatment?

( ) Azathioprine ( ) Cyclosporin ( ) Immunoglobulin ( ) Gamma interferon ( ) Methotrexate

( ) Mofetil Mycophenolate ( ) Omalizumab ( ) Others

19. With Dupilumab available in the Brazilian pharmaceutical market, do you have any patient to start this therapy?

( ) Yes ( ) No

20. Do you usually investigate the association between atopic dermatitis and food allergy?

( ) Always ( ) Almost always ( ) Sometimes ( ) Almost never ( ) Never

21. Do you request specific IgE research for the suspected allergen by which method? (Select one or more alternatives)

( ) Don’t require ( ) Skin test ( ) RAST ( ) PRIST ( ) ImmunoCap® ( ) ImmunoCap ISAC®

22. Do you place dietary restrictions on AD patients?

( ) Yes ( ) No

23. Are dietary restrictions based on positive allergy skin tests/specific IgE test and consistent with clinical history of cause-and-effect relationship?

( ) Yes ( ) No

24. Do you recommend environmental control of aeroallergens to AD patients?

( ) Yes ( ) No

25. Do you investigate immunodeficiencies in patients with moderate/severe AD?

( ) Always ( ) Almost always ( ) Sometimes ( ) Almost never ( ) Never

26. What tests would you order to investigate immunodeficiencies in patients with moderate/severe AD? (Select one or more alternatives)

( ) Complete blood count ( ) Serum immunoglobulin dosage ( ) Lymphocyte subpopulations research ( ) HIV serology ( ) Gene mutation research ( ) Exome ( ) Would not order
